# Availability of Medications for Opioid Use Disorder in Opioid Treatment Programs

**DOI:** 10.1001/jamanetworkopen.2025.17616

**Published:** 2025-06-26

**Authors:** Zoe Lindenfeld, Jonathan H. Cantor, Amanda I. Mauri, Sachini Bandara, Aarya Suryavanshi, Noa Krawczyk

**Affiliations:** 1 Edward J. Bloustein School of Planning and Public Policy, Rutgers University, New Brunswick, New Jersey; 2 RAND, Santa Monica, California; 3 Department of Health Policy and Management, School of Public Health, University of Maryland, College Park; 4 Department of Mental Health, Johns Hopkins Bloomberg School of Public Health, Baltimore, Maryland; 5 Department of Health Policy and Management, Johns Hopkins Bloomberg School of Public Health, Baltimore, Maryland; 6 Department of Population Health, NYU Grossman School of Medicine, New York, New York

## Abstract

**Importance:**

As the primary facilities authorized to dispense methadone, opioid treatment programs (OTPs) are a critical access point for medications for opioid use disorder (MOUD). However, research is limited on the extent to which OTPs offer a broad range of MOUD and on the characteristics of programs that provide more comprehensive medication offerings.

**Objective:**

To assess the percentage of US OTPs offering all 3 forms of MOUD (methadone, buprenorphine, and naltrexone) and compare organizational and county characteristics of OTPs with different MOUD service offerings.

**Design, Setting, and Participants:**

This longitudinal cross-sectional study used data on a panel of OTPs listed in the annual National Directory of Drug and Alcohol Use Treatment Facilities from 2017 to 2023.

**Main Outcomes and Measures:**

Measures included the percentage of OTPs offering buprenorphine, extended-release naltrexone, or all 3 MOUD from 2017 to 2023 (assuming all OTPs offered methadone). Descriptive statistics on organizational and county characteristics of OTPs by MOUD offerings were collected. Three longitudinal logistic regression models were used to estimate the odds of different MOUD offerings within OTPs, adjusting for organizational and county-level characteristics.

**Results:**

This analysis included 10 298 facility-year observations, ranging from 1211 in 2017 to 1421 in 2023. From 2017 to 2023, the percentage of OTPs offering MOUD beyond methadone increased (buprenorphine: 811 [67.0%] in 2017 to 1209 [85.1%] in 2023; naltrexone: 463 [38.2%] in 2017 to 749 [52.7%] in 2023; all 3 MOUD: 402 [33.2%] in 2017 to 639 [45.0%] in 2023). OTPs offering all 3 MOUD (3985 [38.7%]) had significantly higher odds of accepting Medicare (adjusted odds ratio [AOR], 2.14; 95% CI, 1.67-2.74); offering peer services (AOR, 1.63; 95% CI, 1.25-2.12), mental health services (AOR, 2.07; 95% CI, 1.53-2.80), and telemedicine services (AOR, 1.53; 95% CI, 1.22-1.92); and being private nonprofit (AOR, 7.45; 95% CI, 4.67-11.87) or government operated (AOR, 41.83; 95% CI, 19.71-88.75) compared with private for profit.

**Conclusions:**

In this cross-sectional study of OTPs, although the availability of MOUD beyond methadone increased over time, most OTPs still did not offer all 3 forms of MOUD as of 2023. Specific organizational characteristics, such as being government operated and accepting Medicare, were associated with more comprehensive MOUD offerings. Future research should evaluate why OTPs vary in their MOUD offerings.

## Key Points

**Question:**

What percentage of US opioid treatment programs (OTPs) offer all 3 forms of medications for opioid use disorder (MOUD) (methadone, buprenorphine, and naltrexone), and what are characteristics of OTPs with wider MOUD availability?

**Findings:**

In this cross-sectional study of 10 298 facility-year observations from 2017 to 2023, the percentage of OTPs offering all 3 MOUD increased from 33% to 45%. Wider MOUD availability was associated with accepting Medicare, government or nonprofit ownership, and offering mental health services.

**Meaning:**

Despite progress, most OTPs nationwide still did not provide all 3 MOUD options, highlighting the need for increased efforts to encourage comprehensive MOUD offering.

## Teaser

This cross-sectional study evaluates the percentage of opioid treatment programs offering all 3 medications for opioid use disorder (MOUD) (methadone, buprenorphine, and naltrexone) and program characteristics associated with offering a wider variety of MOUD.

## Introduction

Over 107 000 deaths due to drug overdose were reported in the US in 2023, approximately 69% of which were attributed to synthetic opioids, such as fentanyl.^1^ To reduce morbidity and mortality among individuals with opioid use disorder (OUD), the evidence supports expanding use of medications for OUD (MOUD) (ie, methadone, buprenorphine, and naltrexone).^2,3^ For decades, the effectiveness of methadone and buprenorphine has been demonstrated by their association with reductions in overdose deaths^4,5^ and health care utilization, including emergency department admissions and inpatient hospitalizations,^6,7^ among other benefits. Yet, access to and use of MOUD treatment remains low, with only 18% of individuals with OUD receiving MOUD in 2023.^8^

Opioid treatment programs (OTPs) are an important access point to MOUD treatment, as OTP facilities are the only facilities where long-term methadone treatment can be legally accessed. Individuals can access other types of MOUD, such as buprenorphine, through primary care clinics and non-OTP substance use disorder treatment facilities, with estimates suggesting that approximately 70% of individuals seeking MOUD treatment receive MOUD outside of OTPs.^9^ OTPs tend to be open 6 or 7 days a week and, altogether, serve over 400 000 patients in the US on any given day.^10^ With these broad hours of operation and stringent requirements for in-person attendance, OTPs are often a frequent point of contact between patients with OUD and health care services, and their staffing structure (eg, availability of medical personnel) equips them to also integrate other essential MOUD, such as buprenorphine and naltrexone. There are benefits to having multiple MOUD available within OUD treatment settings. Studies have found that MOUD preferences among patients with OUD and health care professionals are diverse, and having a range of options facilitates delivery of services that are truly patient centered.^11,12^ For example, buprenorphine prescriptions or formulations that are administered monthly or every 6 months may be an attractive alternative to OTP rules that require patients to visit OTPs daily for methadone treatment^13,14^ or place limitations on the number of take-home methadone doses a patient can receive.^15^ For other patients, long-acting formulations may represent a reduction in autonomy over treatment decisions.^11,12^ As such, offering a wider range of MOUD options may lead to greater patient-centered care and, therefore, higher initiation and retention of MOUD treatment, particularly in settings that otherwise lack access to buprenorphine prescribers.

While a prior study indicated that MOUD options beyond methadone remained limited in OTPs,^16^ there is limited longitudinal research examining how OTPs have expanded their MOUD offerings over time and the characteristics of programs that provide a broader range of MOUD treatments. One prior study used a survey to understand the characteristics of OTPs, including MOUD availability, organizational characteristics, and additional services offered.^17^ However, that study focused only on differences between for-profit and not-for-profit OTPs. Other studies used surveys of OTPs to assess the availability of services within OTPs after 2020, when Medicare coverage was expanded to include methadone as part of the Substance Use Disorder Prevention That Promotes Opioid Recovery and Treatment for Patients and Communities (SUPPORT) Act.^18,19,20^ However, these studies mainly focused on differences in Medicare acceptance between OTPs based on ownership and for-profit status,^18,19,20^ county-level offering of MOUD,^21^ and changes in OTP utilization following the Medicare policy change.^9,19,22^

The current study aimed to fill existing gaps in research by leveraging a longitudinal panel of OTPs to compare changes in MOUD offerings over time and the organizational and county characteristics of OTPs with different MOUD service offerings. Examining the organizational characteristics and ancillary services offered by OTPs is critical to identify potential facilitators or barriers to expanding the range of MOUD made available to patients and to assess whether OTPs with expanded MOUD offerings are filling service gaps in their communities. Additionally, given that prior research has found reduced access to MOUD and other forms of substance use disorder treatment in lower-income areas^23^ (as well as those with a higher proportion of racial and ethnic minority residents^24,25^), we included several demographic and socioeconomic measures to explore their association with access to OTPs offering additional MOUD. As the landscape of OTP services continues to evolve, this research may help guide decisions about how to best leverage OTPs in responding to the ongoing opioid crisis and variable patient preferences, including decisions related to the optimal service offerings within these facilities.

## Methods

### Study Data

This cross-sectional study used national organization-level data on OTPs from the 2018 to 2024 Mental Health and Addiction Treatment Tracking Repository (MATTR), a national longitudinal database of licensed substance use disorder treatment facilities.^26^ Facilities in the MATTR are respondents to the National Survey of Substance Abuse Treatment Services (N-SSATS) and National Substance Use and Mental Health Services Survey (N-SUMHSS) that agree to be listed in the National Directory of Drug and Alcohol Use Treatment Facilities. The 2018 to 2024 N-SSATS and N-SUMHSS substance use disorder directories present annual data collected from surveys from 2017 to 2023. The Rutgers University institutional review board deemed this study exempt from review, with a waiver of informed consent, because it did not involve human participants. We followed the Strengthening the Reporting of Observational Studies in Epidemiology (STROBE) reporting guideline for cross-sectional studies.

Only facilities that self-reported that they were designated as Substance Abuse and Mental Health Services Administration (SAMHSA)–certified OTPs were included in our main analysis. We organized clinics into 3 categories: OTPs that offered buprenorphine, those that offered extended-release naltrexone, and those that offered all 3 MOUD (methadone, buprenorphine, and naltrexone). Given that the primary purpose of OTPs is to dispense methadone, we assumed all OTPs offered methadone.^15^ Using the full MATTR dataset, we also calculated the proportion of all substance use disorder treatment facilities that were licensed OTPs.

We examined whether several organizational and county-level characteristics were associated with offering different combinations of MOUD. At the organization level, we extracted several covariates from MATTR corresponding to other services offered (ie, peer services, naloxone and overdose education, mental health services, hepatitis education and counseling, and HIV education and counseling), payment options (whether Medicaid and/or Medicare was accepted), facility ownership (nonprofit, for profit, or government), and whether the facility offered telemedicine visit modalities. eTable 1 in Supplement 1 reports how the organizational characteristics included in our study were captured in the N-SUMHSS survey. For county-level characteristics, we used county-level geographic identifiers to merge the MATTR data with data on county characteristics and resources from multiple sources from 2017 to 2023. From the American Community Survey, we extracted population measures for percentages of Black, Hispanic, and White individuals; uninsured individuals; and residents with income under the federal poverty level (FPL).^27^

### Statistical Analysis

We calculated the proportion OTPs that offered buprenorphine, naltrexone, and all 3 MOUD (methadone, buprenorphine, and naltrexone). Within these 3 categories, we used descriptive statistics to estimate the proportion of clinics by our organizational and county-level variables of interest. We also created maps demonstrating the distribution of OTPs offering buprenorphine, naltrexone, and all 3 MOUD within US counties for 2023, the most recent year of available data (eFigure in Supplement 1).

We then conducted 3 longitudinal logistic regression models with facilities clustered at the state level to examine the factors associated with different MOUD offerings within OTPs (model 1: agency offered buprenorphine, whether in combination with methadone only or with all 3 MOUD; model 2: agency offered naltrexone, whether in combination with methadone only or with all 3 MOUD; and model 3: agency offered all 3 MOUD). Each model included organizational and county-level characteristics as variables and an indicator for US census region (Northeast, South, Midwest, or West). We also conducted a sensitivity analysis of our main models that included county-level overdose death rates, drawn from County Health Rankings,^28^ and the number of non-OTP substance use disorder treatment facilities in a county offering buprenorphine per 100 000 residents, sourced from the MATTR database. All analyses were conducted with Stata SE, version 18 (StataCorp LLC). Two-sided *P* < .05 was considered significant.

## Results

This analysis included 10 298 facility-year observations in the MATTR database, ranging from 1211 in 2017 to 1421 in 2023. In total, 8109 of 10 298 OTPs (78.7%) offered buprenorphine, 4702 (45.7%) offered naltrexone, and 3985 (38.7%) offered all 3 MOUD. Among all OTPs, the mean (SD) proportion of Black individuals in the surrounding county was 14.92% (14.13%); Hispanic individuals, 17.34% (15.88%); and White individuals, 67.56% (17.87%). Over the study period, the percentage of OTPs offering all 3 MOUD increased from 33.2% (402 of 1211) in 2017 to 45.0% (639 of 1421) in 2023 (Figure and eTable 2 in Supplement 1). From 2017 to 2023, the percentage of OTPs offering buprenorphine (2017: 811 [67.0%]; 2023: 1209 [85.1%]) and naltrexone (2017: 463 [38.2%]; 2023: 749 [52.7%]) also increased, with buprenorphine offered in more OTPs compared with naltrexone. The percentage of all substance use disorder treatment facilities in the US that were OTPs with different service offerings is reported in eTable 3 in Supplement 1. OTPs accounted for 1421 of all 11 471 substance use disorder treatment facilities (12.4%) in 2023, and the number of OTPs that offered all 3 MOUD in the same year was 639 (4.5%).

**Figure.**
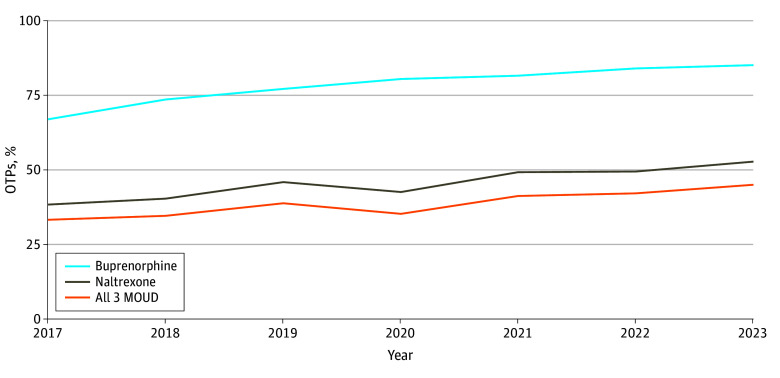
Percentage of Opioid Treatment Programs (OTPs) Offering Medications for Opioid Use Disorder (MOUD), 2017-2023 The total number of OTPs was 10 298. We assumed all OTPs offered methadone given that the primary purpose of OTPs is to dispense methadone.

Descriptive statistics for OTPs with different MOUD offerings are reported in Table 1. OTPs offering buprenorphine had the highest proportion of private for-profit facilities (4814 of 8109 [59.4%]), OTPs offering naltrexone had the highest proportion of private nonprofit facilities (1593 of 4702 [33.9%]), and OTPs offering all 3 MOUD had the highest proportion of government-owned facilities (450 of 3985 [11.3%]). A higher proportion of the 3985 OTPs that offered all 3 MOUD accepted Medicaid (3454 [86.7%]) and Medicare (2621 [65.8%]) compared with OTPs with the other MOUD offerings. A higher proportion of OTPs that offered all 3 MOUD also offered peer services (2284 [57.3%]), naloxone and overdose education services (2467 [61.9%]), and telemedicine services (2197 [55.1%]) compared with OTPs with other MOUD offerings. In contrast, a higher proportion of the 4702 OTPs offering naltrexone provided mental health services (2619 [55.7%]) and hepatitis education and counseling (3522 [74.9%]) compared with OTPs offering buprenorphine or all 3 MOUD.

**Table 1.** Descriptive Statistics Reporting Characteristics of OTPs by MOUD Offering, 2017-2023

Characteristic	Facility-year observations^a^			
	All	Offering buprenorphine	Offering naltrexone	Offering all 3 MOUD
Total	10 298 (100)	8109 (78.7)	4702 (45.7)	3985 (38.7)
OTP characteristics				
Facility operation				
Private for profit	5934 (57.6)	4814 (59.4)	2188 (46.5)	1850 (46.4)
Private nonprofit	2487 (24.2)	1922 (23.7)	1593 (33.9)	1323 (33.2)
Government owned	690 (6.7)	585 (7.2)	498 (10.6)	450 (11.3)
Medicaid accepted	8309 (80.7)	6696 (82.6)	4005 (85.2)	3454 (86.7)
Medicare accepted	5635 (54.7)	4776 (58.9)	2983 (63.4)	2621 (65.8)
Services offered within OTP				
Peer services	4926 (47.8)	4085 (50.4)	2679 (57.0)	2284 (57.3)
Naloxone and overdose education	5886 (57.2)	4882 (60.2)	2906 (61.8)	2467 (61.9)
Mental health services	3865 (37.5)	3288 (40.5)	2619 (55.7)	2195 (55.1)
Hepatitis education and counseling	7635 (74.1)	5938 (73.2)	3522 (74.9)	2973 (74.6)
HIV education and counseling	7651 (74.3)	4667 (57.6)	3467 (73.7)	2919 (73.2)
Telemedicine and/or telehealth therapy	4664 (45.3)	3992 (49.2)	2578 (54.8)	2197 (55.1)
County characteristics				
Race and ethnicity, mean (SD), %				
Black	14.92 (14.13)	14.21 (13.71)	13.57 (12.83)	13.23 (13.01)
Hispanic	17.34 (15.88)	17.16 (15.60)	16.50 (14.43)	16.86 (14.67)
White	67.56 (17.87)	67.99 (17.76)	68.74 (17.56)	68.64 (17.77)
Uninsured individuals, mean (SD), %	8.44 (4.06)	8.32 (3.84)	7.64 (3.63)	7.67 (3.60)
Households with income <FPL, mean (SD), %	9.89 (4.24)	9.69 (4.12)	9.39 (4.05)	9.41 (4.10)

Abbreviations: FPL, federal poverty level; MOUD, medications for opioid use disorder; OTP, opioid treatment program.

^a^ Data are presented as number (percentage) of facility-year observations unless otherwise indicated.

Table 2 reports the results of our longitudinal logistic regression model estimating the availability of MOUD within OTPs. OTPs that offered all 3 MOUD had significantly higher odds of accepting Medicare (adjusted odds ratio [AOR], 2.14; 95% CI, 1.67-2.74); offering peer services (AOR, 1.63; 95% CI, 1.25-2.12), mental health services (AOR, 2.07; 95% CI, 1.53-2.80), and telemedicine services (AOR, 1.53; 95% CI, 1.22-1.92); and being private nonprofit (AOR, 7.45; 95% CI, 4.67-11.87) or government owned (AOR, 41.83; 95% CI, 19.71-88.75) compared with private for profit. They also had significantly lower odds of offering HIV education and counseling (AOR, 0.49; 95% CI, 0.32-0.77), being located in a county with a higher percentage of households with income less than the FPL (AOR, 0.93; 95% CI, 0.88-0.98), and being located in the South region of the US compared with the Northeast (AOR, 0.36; 95% CI, 0.19-0.69). OTPs that offered buprenorphine had significantly higher odds of accepting Medicaid (AOR, 2.01; 95% CI, 1.15-3.48) and Medicare (AOR, 2.63; 95% CI, 1.81-3.80), offering peer services (AOR, 2.41; 95% CI, 1.59-3.61) and telemedicine services (AOR, 3.91; 95% CI, 2.73-5.61), and being located in the South (AOR, 8.54; 95% CI, 3.30-22.05) or West (AOR, 24.64; 95% CI, 9.09-66.80) compared with the Northeast. OTPs with buprenorphine had significantly lower odds of offering HIV counseling and education (AOR, 0.50; 95% CI, 0.26-0.95), being located in a county with a higher percentage of households with income less than the FPL (AOR, 0.88; 95% CI, 0.82-0.95) and uninsured residents (AOR, 0.85; 95% CI, 0.76-0.96), and being a private nonprofit facility compared with a private for-profit facility (AOR, 0.35; 95% CI, 0.18-0.68). OTPs with naltrexone had significantly higher odds of accepting Medicare (AOR, 1.71; 95% CI, 1.33-2.18), offering peer services (AOR, 1.62; 95% CI, 1.25-2.10) and mental health services (AOR, 3.13; 95% CI, 2.31-4.23), and being private nonprofit (AOR, 8.42; 95% CI, 5.37-13.20) or government owned (AOR, 39.67; 95% CI, 19.19-82.00) compared with private for-profit and had significantly lower odds of offering HIV education and counseling (AOR, 0.44; 95% CI, 0.29-0.69), being located in a county with a higher percentage of households with income less than the FPL (AOR, 0.91; 95% CI, 0.87-0.96), and being located in the South region of the US compared with the Northeast (AOR, 0.19; 95% CI, 0.10-0.36). Results from models that included county-level overdose death rates and non-OTP buprenorphine treatment availability from substance use disorder treatment facilities were consistent with our main analyses (eTable 4 in Supplement 1). Results from models with OTP-service variables omitted can be found in eTable 5 in Supplement 1.

**Table 2.** Logistic Regression Models Estimating Odds of Different MOUD Availability by OTP Variables

Variable	AOR (95% CI)^a^		
	Buprenorphine (n = 9073)	Naltrexone (n = 9073)	All 3 MOUD (n = 9073)
Facility operation			
Private for profit	1 [Reference]	1 [Reference]	1 [Reference]
Private nonprofit	0.35 (0.18-0.68)^b^	8.42 (5.37-13.20)^b^	7.45 (4.67-11.87)^b^
Government owned	2.61 (0.81-8.37)	39.67 (19.19-82.00)^b^	41.83 (19.71-88.75)^b^
Medicaid accepted	2.01 (1.15-3.48)^c^	0.65 (0.45-0.94)^c^	0.75 (0.51-1.09)
Medicare accepted	2.63 (1.81-3.80)^b^	1.71 (1.33-2.18)^b^	2.14 (1.67-2.74)^b^
Peer services	2.41 (1.59-3.61)^b^	1.62 (1.25-2.10)^b^	1.63 (1.25-2.12)^b^
Naloxone and overdose education	1.37 (0.98-1.92)	1.01 (0.80-1.27)	1.05 (0.83-1.32)
Mental health services	1.77 (1.11-2.83)^c^	3.13 (2.31-4.23)^b^	2.07 (1.53-2.80)^b^
Hepatitis education and counseling	0.71 (0.37-1.36)	0.91 (0.60-1.39)	0.85 (0.55-1.30)
HIV education and counseling	0.50 (0.26-0.95)^c^	0.44 (0.29-0.69)^b^	0.49 (0.32-0.77)^b^
Telemedicine and/or telehealth therapy	3.91 (2.73-5.61)^b^	1.77 (1.41-2.22)^b^	1.53 (1.22-1.92)^b^
Higher percentage of White individuals in county	0.99 (0.97-1.01)	1.00 (0.98-1.01)	1.00 (0.98-1.01)
Higher percentage of uninsured individuals in county	0.85 (0.76-0.96)^b^	0.96 (0.91-1.02)	0.95 (0.89-1.01)
Higher percentage of households with income <FPL in county	0.88 (0.82-0.95)^b^	0.91 (0.87-0.96)^b^	0.93 (0.88-0.98)^c^
Region			
Northeast	1 [Reference]	1 [Reference]	1 [Reference]
South	8.54 (3.30-22.05)^b^	0.19 (0.10-0.36)^b^	0.36 (0.19-0.69)^b^
Midwest	1.65 (0.62-4.36)	1.34 (0.71-2.51)	1.01 (0.52-1.94)
West	24.64 (9.09-66.80)^b^	0.45 (0.24-0.86)^c^	1.68 (0.88-3.23)

Abbreviations: AOR, adjusted odds ratio; FPL, federal poverty level; MOUD, medications for opioid use disorder; OTP, opioid treatment program..

^a^ Adjusted for organizational and county-level characteristics.

^b^ *P* < .01.

^c^ *P* < .05.

## Discussion

This longitudinal cross-sectional study of OTPs in the US found that most programs still did not offer all 3 forms of MOUD as of 2023. Given that OTPs are the only facilities in which methadone can be legally dispensed and that OTPs accounted for 12.4% of all substance use disorder treatment facilities in 2023, the number of OTPs that offered all 3 MOUD in the same year was small (4.5%). However, it is notable that availability of both buprenorphine and naltrexone within these facilities increased over time and their availability was associated with particular organizational factors and county-level characteristics. There was also an increase in the number of OTPs in our study sample over the study period, broadening the availability of methadone.

Accepting Medicare as a form of payment was associated with greater availability of all 3 MOUD, while accepting Medicaid was only associated with higher availability of buprenorphine. In January 2020, Medicare began covering care received at OTPs for the first time since its inception. Medicare pays for OTP care using weekly bundled payments that cover care management, counseling, therapy, and toxicology testing in addition to MOUD.^29^ In contrast, Medicaid coverage of methadone differs between states, and the average weekly Medicaid bundled fee is substantially lower than the weekly Medicare fee.^30^ Prior studies conducted since 2020 found that accepting Medicare as a form of payment was associated with receiving forms of accreditation that indicate an OTP provides higher-quality care^20^ and with increases in the share of Medicare beneficiaries treated at OTPs.^9^ Further research should evaluate what organizational, geographic, and patient characteristics cause OTPs to accept certain payment forms over others and to offer additional MOUD.

Fewer OTPs offered naltrexone compared with buprenorphine. In part, this may be due to fewer patients seeking or being eligible for naltrexone given that they must be abstinent from all forms of opioids for 7 to 10 days prior to receiving their first dose and remain abstinent throughout their treatment course.^31^ A previous survey of OTPs found that 48% cited clinical logistical concerns with naltrexone induction as a reason for not offering naltrexone within their facility.^17^ Beyond induction-related concerns, within the addiction medicine community, naltrexone is not favored as a first-line MOUD treatment,^32^ as it may not adequately treat opioid cravings^33^ and is associated with higher disengagement from treatment and worsened clinical outcomes compared with other MOUD.^13^ As such, it may be less critical for OTPs to offer naltrexone compared with buprenorphine and methadone. However, OTPs that offered naltrexone also had significantly higher odds of offering mental health services. A potential hypothesis based on these results is that OTPs that succeed in overcoming initial dispensing challenges are those with a preexisting infrastructure to support services that may require more dedicated staff time and resources. Additionally, naltrexone has a significantly higher overall cost, driven by both the longer detoxification period required for initiation and the higher price of the medication. One study found that naltrexone costs the health care system an average of $5317 more per patient than buprenorphine.^34^ Previous qualitative research has listed cost as a barrier for naltrexone to be offered by substance use disorder treatment centers.^35^ Our finding that nonprofit and government-operated OTPs had significantly higher odds of offering both naltrexone and all 3 MOUD compared with for-profit OTPs may lend support for this hypothesis, as a greater focus on revenue within for-profit facilities may deincentivize them from offering more costly and time-intensive services.

Our results indicate potential geographic disparities in the availability of additional MOUD in OTPs. Specifically, OTPs located in counties with a higher percentage of residents with income less than the FPL and uninsured residents had significantly lower odds of offering additional MOUD. In part, this may be due to lower rates of reimbursement and payment in these areas. We also found mixed results pertaining to MOUD offerings and geographic region, with OTPs in the South being significantly more likely to offer buprenorphine than Northeast OTPs but significantly less likely to offer naltrexone and all 3 MOUD. Regional differences in reimbursement for MOUD may in part underlie these results.^36^ However, our finding pertaining to buprenorphine indicates that OTPs may be critical access points for this medication in the South region of the US, particularly as other studies have documented limited availability of health care professionals providing buprenorphine in this region.^37,38^ Specifically, our results suggest that OTPs may have the potential to compensate buprenorphine prescriber shortages in these areas, as OTPs may have greater capacity for large volumes of patients with OUD compared with office-based opioid treatment facilities and may be eligible for more flexible telehealth practices than practitioners providing care in clinics.^39^

### Limitations

A key limitation of this study is the reliance on survey response data, which may not accurately reflect actual prescribing and dispensing practices due to desirability bias; for example, facilities may have overstated their capability to provide all forms of MOUD, and as such, the study’s results should be confirmed using claims data reflecting buprenorphine prescribing and dispensing rates. Additionally, not all OTPs are included in the MATTR dataset, which only includes OTPs that responded to the SAMHSA N-SSATS and N-SUMHSS and agreed to be listed in the annual national directories. As such, OTPs that did not complete the N-SSATS or N-SUMHSS or did not agree to be listed in the directory were not included in this analysis. Staff capacity and patient volume are likely important drivers of whether an OTP offers additional MOUD; however, the data used for this analysis did not include these measures. Similarly, we had no information about the quality of care provided by the OTPs and did not include other geographic measures, such as urban density, the number of health care professionals that offered or used buprenorphine, and the availability of specialized health care professionals. In addition, given our cross-sectional study design, we are unable to make causal inferences between factors examined and our outcome of interest.

## Conclusions

This cross-sectional study is an important contribution to the literature on OTPs, revealing that nonprofit and government-operated OTPs and OTPs that accepted Medicare had a greater likelihood of offering all 3 MOUD. Despite these facilitators, only 45.0% of OTPs offered all 3 MOUD as of 2023, signaling that additional effort, such as financial incentives or staff training, may be needed to further expand availability of a wider range of MOUD within OTPs, particularly in communities that have historically had reduced access to buprenorphine treatment.

## Notes

Supplement 1. **eTable 1.** Organizational Characteristics Captured in the N-SHUMSS and N-SSATS Service Codes **eTable 2.** Number of OTPs by MOUD Offering Each Year **eTable 3.** Proportion of All Substance Use Treatment Facilities That Were OTPs, 2017-2023 **eTable 4.** Logistic Regression Models Estimating OTPs With Different MOUD Availability, Controlling for Overdose Crisis Severity and Buprenorphine Availability at the County Level **eTable 5.** Logistic Regression Models Estimating OTPs With Different MOUD Availability, Without Service-Related Variables **eFigure.** Opioid Treatment Programs Offering Buprenorphine, Naltrexone, and All 3 MOUD Within US Counties, 2023

Supplement 2.
Data Sharing Statement
