# Supplementary material for: Availability of Medications for Opioid Use Disorder in Opioid Treatment Programs
Source: JAMA Netw Open. 2025 Jun 26;8(6):e2517616. doi: 10.1001/jamanetworkopen.2025.17616 (PMC12203276; doi:10.1001/jamanetworkopen.2025.17616)
Supplement: Supplement 1. — eTable 1. Organizational Characteristics Captured in the N-SHUMSS and N-SSATS Service Codes eTable 2. Number of OTPs by MOUD Offering Each Year eTable 3. Proportion of All Substance Use Treatment Facilities That Were OTPs, 2017-2023 eTable 4. Logistic Regression Models Estimating OTPs With Different MOUD Availability, Controlling for Overdose Crisis Severity and Buprenorphine Availability at the County Level eTable 5. Logistic Regression Models Estimating OTPs With Different MOUD Availability, Without Service-Related Variables eFigure. Opioid Treatment Programs Offering Buprenorphine, Naltrexone, and All 3 MOUD Within US Counties, 2023 [file jamanetwopen-e2517616-s001.pdf]

Supplementary Online Content

Lindenfeld Z, Cantor JH, Mauri AI, Bandara S, Suryavanshi A, Krawczyk N. Availability of medications for opioid use disorder in opioid treatment programs. *JAMA Netw Open*. 2025;8(6):e2517616. doi:10.1001/jamanetworkopen.2025.17616

eTable 1. Organizational Characteristics Captured in the N-SHUMSS and N-SSATS Service Codes
eTable 2. Number of OTPs by MOUD Offering Each Year
eTable 3. Proportion of All Substance Use Treatment Facilities That Were OTPs, 2017-2023
eTable 4. Logistic Regression Models Estimating OTPs With Different MOUD Availability, Controlling for Overdose Crisis Severity and Buprenorphine Availability at the County Level
eTable 5. Logistic Regression Models Estimating OTPs With Different MOUD Availability, Without Service-Related Variables
eFigure. Opioid Treatment Programs Offering Buprenorphine, Naltrexone, and All 3 MOUD Within US Counties, 2023

This supplementary material has been provided by the authors to give readers additional information about their work.

© 2025 Lindenfeld Z et al. *JAMA Network Open*.

eTable 1. Organizational characteristics included in our study captured in the N-SHUMSS and N-SSATS service codes

Organizational Characteristics	Service Codes
OTP Certification	SOTP
Naltrexone offered	UN, NU
Buprenorphine offered	UB, BU
Private for-profit facility	PVTP
Private non-profit facility	PVTN
Government-operated	LCCG, STG, FED
Medicaid accepted	MD
Medicare accepted	MC
Peer Services	PEER
Naloxone and overdose education	NOE
Mental health services	MHS
Hepatitis education and counseling	TAEC
HIV education and counseling	HAEC
Telemedicine/telehealth therapy	TELE

© 2025 Lindenfeld Z et al. *JAMA Network Open*.

eTable 2. Number of OTPs by MOUD offering each year

Year	Methadone only	Methadone + Buprenorphine (alone or in combination with naltrexone)	Methadone + Buprenorphine Only	Methadone + Naltrexone (alone or in combination with buprenorphine)	Methadone + Naltrexone Only	Methadone + Buprenorphine + Naltrexone
2017	361 (29.80)	811 (66.97)	387 (31.96)	463 (38.23)	39 (3.22)	402 (33.20)
2018	332 (23.60)	1,036 (73.63)	507 (36.03)	568 (40.37)	39 (2.77)	487 (34.61)
2019	307 (19.88)	1,191 (77.14)	529 (34.26)	708 (45.85)	46 (2.98)	597 (38.67)
2020	275 (17.16)	1,288 (80.35)	646 (40.30)	682 (42.55)	40 (2.50)	565 (35.25)
2021	238 (15.24)	1,272 (81.43)	558 (35.72)	766 (49.04)	52 (3.33)	644 (41.23)
2022	212 (13.68)	1,302 (84.00)	572 (36.90)	766 (49.42)	36 (2.32)	651 (42.00)
2023	181 (12.74)	1,209 (85.08)	491 (34.55)	749 (52.71)	31 (2.18)	639 (44.97)

© 2025 Lindenfeld Z et al. *JAMA Network Open*.

eTable 3. Percentage of all substance use treatment facilities that were OTPs, 2017-2023

Year	Number of SUD Treatment Facilities	Percentage of SUD Treatment Facilities that are OTPS	Percentage of SUD Treatment Facilities that offer all three MOUD
2017	13,240	9.15% (1,211)	3.03% (402)
2018	13,886	10.1% (1,407)	3.51% (487)
2019	14,341	10.8% (1,544)	4.16% (597)
2020	14,229	11.4% (1,603)	3.97% (565)
2021	12,981	12.0% (1,562)	4.99% (644)
2022	12,704	12.2% (1,550)	5.12% (651)
2023	11,471	12.4% (1,421)	5.57% (639)

© 2025 Lindenfeld Z et al. *JAMA Network Open*.

eTable 4. Logistic regression models predicting OTPs with different MOUD availability, controlling for overdose crisis severity and buprenorphine availability at the county level (reports odds ratios with 95% confidence intervals)

Variable	Outcome:OTPs with Buprenorphine (n=7,662)	Outcome:OTPs with Naltrexone (n=7,662)	Outcome:OTPs with all three MOUD (n=7,662)
**Facility Operation**			
Private for-profit	*Base Category*	*Base Category*	*Base Category*
Private non-profit	0.21 (0.98-0.47)**	6.23 (3.79-10.22)**	4.27 (2.56-7.13)**
Government-owned	1.44 (0.40-5.22)	27.59 (12.66-60.13)**	22.46 (10.03-50.30)**
**Medicaid accepted**	2.20 (1.15-4.20)*	0.65 (0.43-0.98)*	0.79 (0.51-1.20)
**Medicare accepted**	2.00 (1.32-3.04)**	1.60 (2.22-2.09)**	1.84 (1.40-2.41)**
**Peer services**	2.97 (1.82-4.84)**	2.66 (1.99-3.567)**	2.55 (1.89-3.44)**
**Naloxone and overdose education**	2.34 (1.56-3.53)**	2.30 (1.75-3.02)**	2.13 (1.61-2.81)**
**Mental health services**	3.79 (2.08-6.90)**	7.25 (5.07-10.35)**	4.90 (3.42-7.02)**
**Hepatitis education and counseling**	1.21 (0.56-2.61)	1.92 (1.17-3.13)**	1.68 (1.02-2.78)*
**HIV education and counseling**	1.13 (0.51-2.50)	0.62 (0.37-1.02)	0.69 (0.41-1.14)
**Telemedicine/telehealth therapy**	3.15 (2.06-4.81)**	1.07 (0.83-1.38)	1.01 (0.78-1.31)
**Percent White in surrounding county**	1.00 (0.98-1.02)	1.02 (1.01-1.03)	1.01 (1.00-1.03)**
**Percent Uninsured in surrounding county**	0.91 (0.80-1.05)	0.97 (0.91-1.04)	0.96 (0.89-1.03)
**Percent of households with incomes under the federal poverty limit in surrounding county**	0.91 (0.83-0.99)*	0.96 (0.911-1.02)	0.98 (0.92-1.04)
**Overdose death rate**	0.99 (0.99-1.00)	0.99 (0.99-1.00)	0.99 (0.99-1.00)
**Number of non-OTP substance use treatment facilities offering buprenorphine per 100,000 residents**	1.07 (0.83-1.38)	0.92 (0.79-1.07)	0.89 (0.76-1.04)

© 2025 Lindenfeld Z et al. *JAMA Network Open*.

**Region**			
Northeast	*Base Category*	*Base Category*	*Base Category*
South	10.04 (3.35-30.04)**	0.27 (0.13-0.54)**	0.44 (0.21-0.88)*
Midwest	1.62 (0.48-5.47)	1.34 (0.69-2.62)	0.89 (0.44-1.79)
West	30.38 (9.71-94.97)**	0.53 (0.28-1.05)	1.86 (0.92-3.76)

*p<0.05;**p<0.01

© 2025 Lindenfeld Z et al. *JAMA Network Open*.

eTable 5. Logistic regression models estimating OTPs with different MOUD availability, without service-related variables (reports odds ratios with 95% confidence intervals)

Variable	Outcome:OTPs with Buprenorphine (n=9,073)	Outcome:OTPs with Naltrexone (n=9,073)	Outcome:OTPs with all three MOUD (n=9,073)
**Facility Operation**			
Private for-profit	*Base Category*	*Base Category*	*Base Category*
Private non-profit	0.35 (0.18-0.66)**	13.79 (8.38-22.69)**	10.12 (6.16-16.61)**
Government-owned	2.45 (0.75-80.1)	84.71 (38.66-185.58)**	67.39 (30.42-149.31)**
**Medicaid accepted**	1.74 (1.01-3.00)*	0.63 (0.43-0.92)*	0.71 (0.49-1.05)
**Medicare accepted**	4.93 (3.50-6.96)**	2.29 (1.82-2.90)**	2.68 (2.23-3.40)
**Percent White in surrounding county**	0.97 (0.95-0.98)**	0.99 (0.98-1.00)	0.99 (0.98-1.01)
**Percent Uninsured in surrounding county**	0.82 (0.73-0.92)**	0.95 (0.89-1.02)	0.94 (0.88-1.01)
**Percent of households with incomes under the federal poverty limit in surrounding county**	0.81 (0.75-0.87)**	0.88 (0.83-0.93)**	0.90 (0.85-0.95)**
**Region**			
Northeast	*Base Category*	*Base Category*	*Base Category*
South	8.39 (3.22-21.80)	0.12 (0.05-0.25)**	0.30 (0.15-0.60)**
Midwest	2.06 (0.76-5.58)	1.47 (0.71-3.05)	1.09 (0.53-2.23)
West	22.46 (8.22-61.35)**	0.36 (0.17-0.75)**	1.68 (0.82-3.44)

*p<0.05;**p<0.01

© 2025 Lindenfeld Z et al. *JAMA Network Open*.

eFigure. Opioid treatment programs offering buprenorphine, naltrexone, and all three MOUD within US counties, 2023*

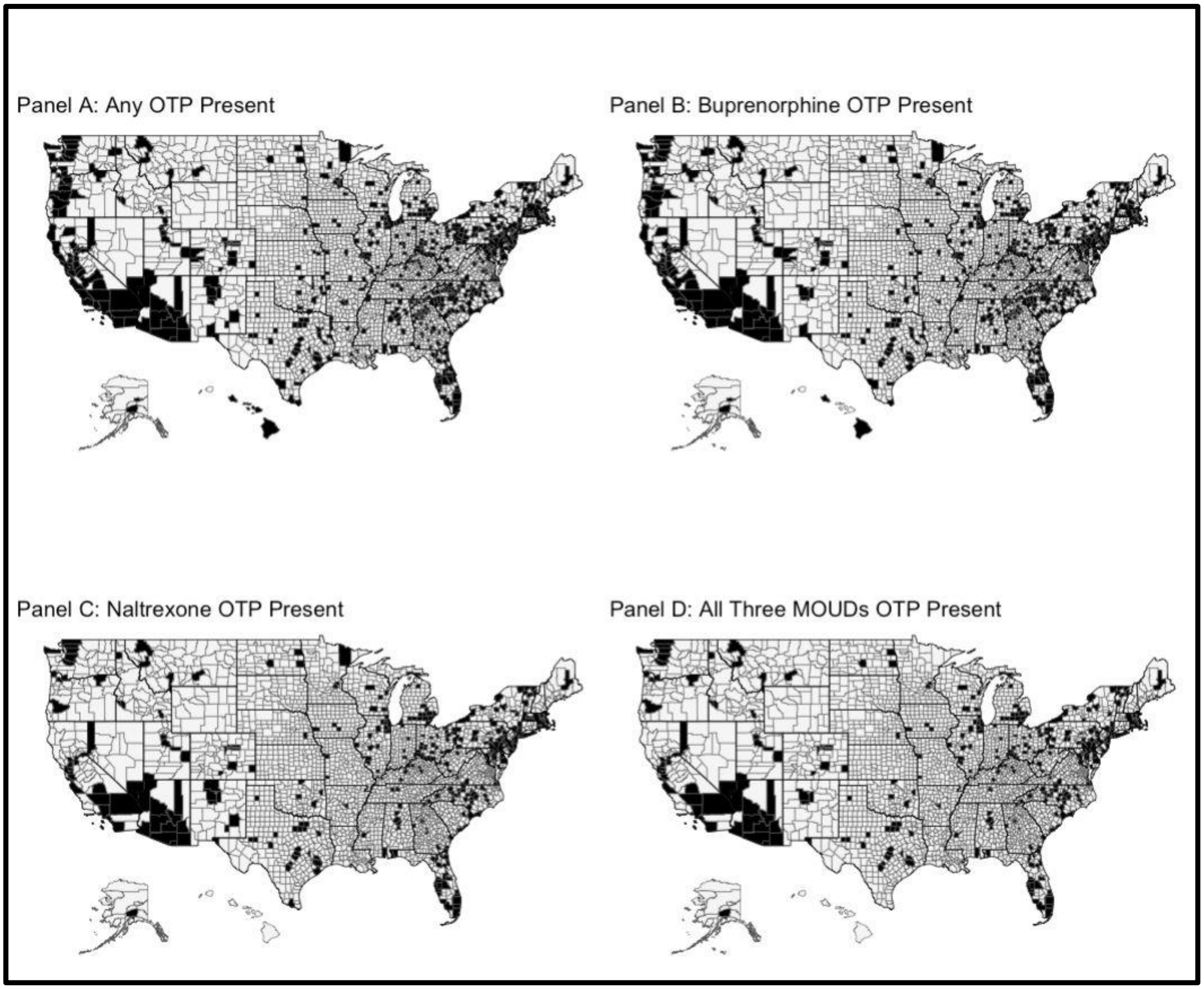

*Black counties indicate the presence of opioid treatment programs with different MOUD offerings; white counties indicate the absence of opioid treatment programs with different MOUD offerings

© 2025 Lindenfeld Z et al. *JAMA Network Open*.
