# Supplementary material for: Availability of Medications for Opioid Use Disorder in Opioid Treatment Programs
Source: JAMA Netw Open. 2025 Jun 26;8(6):e2517616. doi: 10.1001/jamanetworkopen.2025.17616 (PMC12203276; doi:10.1001/jamanetworkopen.2025.17616)
Supplement: Supplement 2. — Data Sharing Statement [file jamanetwopen-e2517616-s002.pdf]

Data Sharing Statement

Lindenfeld. Availability of Medications for Opioid Use Disorder in Opioid Treatment Programs. *JAMA Netw Open*. Published June 26, 2025. doi:10.1001/jamanetworkopen.2025.17616

Data
Data available: No

Additional Information
Explanation for why data not available: This study uses publicly available data on substance use treatment facilities.
